# Feasibility of Using a Smartwatch to Intensively Monitor Patients With Chronic Obstructive Pulmonary Disease: Prospective Cohort Study

**DOI:** 10.2196/10046

**Published:** 2018-06-14

**Authors:** Robert Wu, Daniyal Liaqat, Eyal de Lara, Tatiana Son, Frank Rudzicz, Hisham Alshaer, Pegah Abed-Esfahani, Andrea S Gershon

**Affiliations:** ^1^ Division of General Internal Medicine University Health Network Toronto, ON Canada; ^2^ Department of Medicine University of Toronto Toronto, ON Canada; ^3^ Department of Computer Science University of Toronto Toronto, ON Canada; ^4^ Toronto Rehabilitation Institute University Health Network Toronto, ON Canada; ^5^ Sunnybrook Research Institute Sunnybrook Health Sciences Centre Toronto, ON Canada; ^6^ Institute for Clinical Evaluative Sciences Toronto, ON Canada

**Keywords:** chronic obstructive pulmonary disease, monitoring, physiologic disease management, wearable, telehealth

## Abstract

**Background:**

Acute exacerbations of chronic obstructive pulmonary disease (COPD) are associated with accelerated decline in lung function, diminished quality of life, and higher mortality. Proactively monitoring patients for early signs of an exacerbation and treating them early could prevent these outcomes. The emergence of affordable wearable technology allows for nearly continuous monitoring of heart rate and physical activity as well as recording of audio which can detect features such as coughing. These signals may be able to be used with predictive analytics to detect early exacerbations. Prior to full development, however, it is important to determine the feasibility of using wearable devices such as smartwatches to intensively monitor patients with COPD.

**Objective:**

We conducted a feasibility study to determine if patients with COPD would wear and maintain a smartwatch consistently and whether they would reliably collect and transmit sensor data.

**Methods:**

Patients with COPD were recruited from 3 hospitals and were provided with a smartwatch that recorded audio, heart rate, and accelerations. They were asked to wear and charge it daily for 90 days. They were also asked to complete a daily symptom diary. At the end of the study period, participants were asked what would motivate them to regularly use a wearable for monitoring of their COPD.

**Results:**

Of 28 patients enrolled, 16 participants completed the full 90 days. The average age of participants was 68.5 years, and 36% (10/28) were women. Survey, heart rate, and activity data were available for an average of 64.5, 65.1, and 60.2 days respectively. Technical issues caused heart rate and activity data to be unavailable for approximately 13 and 17 days, respectively. Feedback provided by participants indicated that they wanted to actively engage with the smartwatch and receive feedback about their activity, heart rate, and how to better manage their COPD.

**Conclusions:**

Some patients with COPD will wear and maintain smartwatches that passively monitor audio, heart rate, and physical activity, and wearables were able to reliably capture near-continuous patient data. Further work is necessary to increase acceptability and improve the patient experience.

## Introduction

Chronic obstructive pulmonary disease (COPD) affects 251 million people worldwide. In 2015, it was estimated to have caused 3.17 million deaths, 5% of all deaths globally [1]. People with COPD have exacerbations or episodes when their breathing, cough, or sputum production worsens, and treatment is warranted. Such acute exacerbations of COPD (AECOPDs) accelerate the decline in lung function, diminish quality of life, and lead to death [2-8]. Early treatment can reduce the severity of AECOPD and prevent hospitalizations, reduce morbidity, and likely reduce mortality [4,9].

Early treatment of AECOPD can be provided with early detection. Patients may be able to detect exacerbations earlier with information from frequent or continuous monitoring of physiologic parameters such as heart rate, respiratory rate, coughing, oxygen saturation, and physical activity. A recent systematic review examined the effectiveness of home telemonitoring for predicting an AECOPD [10]. Of the 16 studies that evaluated the predictive ability of systems that recorded physiologic parameters or symptoms, none appeared to be clinically reliable in predicting AECOPDs. One reason may be that most data are collected too infrequently. Most systems collected data once daily, potentially missing the sensitivity required to detect early AECOPDs [10].

The emergence of consumer wearables such as smartwatches offers a potential practical and affordable method to monitor and collect early signs frequently, even continuously, to detect concerning symptoms. However, such devices can only work if they are worn and maintained. While elderly patients have said they would be willing to use telemonitoring devices to improve their care [11], studies have found recruitment difficulties, with up to 80% refusing to use the device and low patient adherence [12,13]. The difficulty in reliably measuring physiologic data has been another reason attributed to the inability of telemonitoring to predict AECOPDs [10].

Our overall goal is to improve the care of people with COPD and reduce hospitalizations by providing patients with a practical way to monitor their condition to detect early exacerbations, allowing for timely intervention. In this study, we sought to determine whether patients with COPD would use, wear, and maintain a smartwatch for extended periods of time and whether such a device could reliably capture near-continuous sensor data.

## Methods

### Patient Recruitment

Participants with COPD aged 40 years and older were recruited from 3 hospitals—Sunnybrook Health Sciences Centre, Toronto General Hospital, and Toronto Western Hospital—and from 3 sources: hospital inpatient wards, respirology clinics, and responses to posters soliciting people with COPD. We excluded those who could not speak English, those who resided in a long-term care facility, those who had a medical condition that impaired their ability to participate in the study, and those who did not provide informed consent. Ethical approval was obtained from the University Health Network and Sunnybrook Health Sciences Centre research ethics committees.

### Intervention

Our wearable system consisted of 3 main components: Android Wear smartwatch, Android phone, and remote server. Participants were provided with instructions on the use and charging of the watch and the phone and how to fill out the symptom survey. The smartwatch collected sensor data that included audio from a microphone on the watch, heart rate, accelerometer, and gyroscope recordings. To avoid depleting the battery, the smartwatch recorded 2 out of every 10 minutes. This strategy resulted in an average battery life of 16 hours, which proved sufficient for a full day’s use. The accelerometer and gyroscope were sampled at 20 Hz. We used 2 smartwatch models: the LG Watch Urbane W150 (LG Electronics) and the Moto 360 2nd Generation (Motorola Mobility LLC; all 42 mm variations, including the sport and women’s models), both running Android 6.0.1. The phone acted as a relay between the smartwatch and server and also prompted the user to fill out the symptom survey. We used LG Nexus 5 (LG Electronics; Android 6.0.1) or Moto G 3rd Generation (Motorola Mobility LLC; Android 6.0) phones. Each mobile phone is equipped with a 5 GB per month data plan, and our recording frequency and sampling rates were selected to fit within this limit. Phones were secured to prevent installation of other apps. The smartwatch sent sensor information to the mobile phone. These data were sent encrypted to a secure server, stored, and later made available for processing and analysis.

### Outcomes

The primary outcome was whether patients with COPD were able to wear and maintain the smartwatches for 90 days. Secondary outcomes included the availability of heart rate, activity, and daily symptom surveys and qualitative feedback from participants at the end of the study. The heart rate and accelerometer data were collected directly from the smartwatches. Participants were also asked to complete a validated London COPD cohort daily symptom questionnaire consisting of 8 questions on the following symptoms: increased breathlessness, increased sputum color, increased sputum amount, a cold (such as runny or blocked nose), increased wheeze or chest tightness, sore throat, increased cough, and fever [3,14]. In accordance with the previous definitions, symptoms were classified as major (dyspnea, sputum purulence, and sputum volume) or minor (nasal discharge/congestion, wheeze, sore throat, cough, and fever). This survey has been validated to identify AECOPDs [3]. Patients who either dropped out or completed the study were also asked for their feedback on the wearable system, how they would want to interact with the system, and how the system could be improved.

### Data Analysis

User behavior was analyzed to determine whether the participants filled out the daily questionnaires on the mobile phone. Questionnaires were scored using the previously validated scoring process to determine the occurrences of AECOPDs [14]. To calculate the daily symptom score, each major symptom was weighted 5 points and minor symptoms were 1 point each. AECOPD was defined when the participant had a score greater than 5 for 2 consecutive days. Resolution of AECOPD occurred when the score was at 0 for 5 days.

Heart rate and accelerometer data were only considered if the device was not charging and their sensor status reading was reported as reliable. Audio data were collected and processed to detect coughing but will be described in another paper. We applied previously established methods to process raw accelerometer sensor data to determine sedentary behavior and physical activity [15]. Initially, raw data were converted to a motion summary count using an area under the curve calculation after removing the effects of gravity, which provided activity counts per minute. Thresholds for sedentary behavior (<50.92 counts per minute) and moderate-vigorous physical activity (>305.36 counts per minute) were used from a validation study of Android devices [16]. Python and the pandas, NumPy, and Scikit libraries were used for analysis.

Two authors (TS and RW) first independently analyzed participant feedback to look for common themes and then met and agreed upon common themes.

## Results

### Patient Recruitment

**Figure 1:**
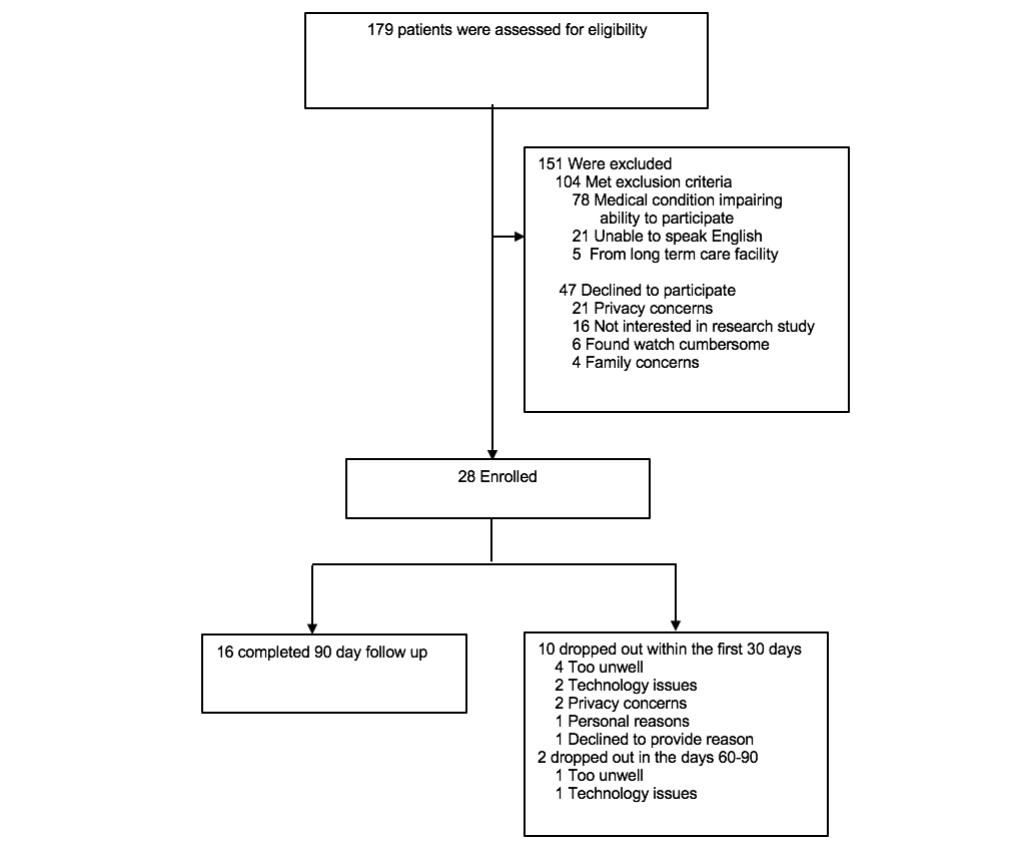
Enrollment and outcomes.

Out of 179 approached, 28 patients were recruited. People were primarily excluded for having a medical condition that impaired their ability to participate or being unable to speak English (Figure 1). The main reasons that eligible patients declined participation included privacy concerns and that they were uninterested in participating in a research study. Of the 28 enrolled patients, 16 completed the 90-day follow-up. The major reasons for dropping out of the study were that they were too sick (5 patients), they experienced technology issues (2), and they had privacy concerns (2). The baseline characteristics are shown in Table 1. Of the 28 participants, the average age was 68.5 (range 41 to 84) years, and 10 participants were women. For the 16 participants who completed follow-up, the average age was 69.3 (range 52-84) years, and 4 were women.

**Table 1:** Patient demographics.

Characteristics	All participants (n=28)	Participants who completed 90 days (n=16)
Age, years, mean	68.5	69.3
Women, %	35	25
FEV_1_^a^/FVC^b^, %	53	56
FEV_1_, %	57	63

^a^FEV_1_: forced expiratory volume in 1 second.

^b^FVC: forced vital capacity.

### Intervention

There were several significant technical issues that occurred and were resolved in the initial 6 months. These issues prevented data to be uploaded and displayed errors on the watch. This caused 3 people to drop out as well as heart rate and accelerometer data to be unavailable for approximately 13 and 17 days, respectively. Other technical issues occurred intermittently in some smartwatches and mobile phones throughout the study period.

### Outcomes

The daily survey questionnaires were completed an average of 47.5% of the time for all participants. For participants who completed the study, surveys were completed 71.7% of the time (range 21.1% to 100%), and the scoring of these surveys indicated 24 exacerbations which lasted an average of 32 (SD 24) days. There was a large variation in how patients scored their symptoms; 6 of 16 patients were considered to be in exacerbation for more than 75% of the time monitored.

**Figure 2:**
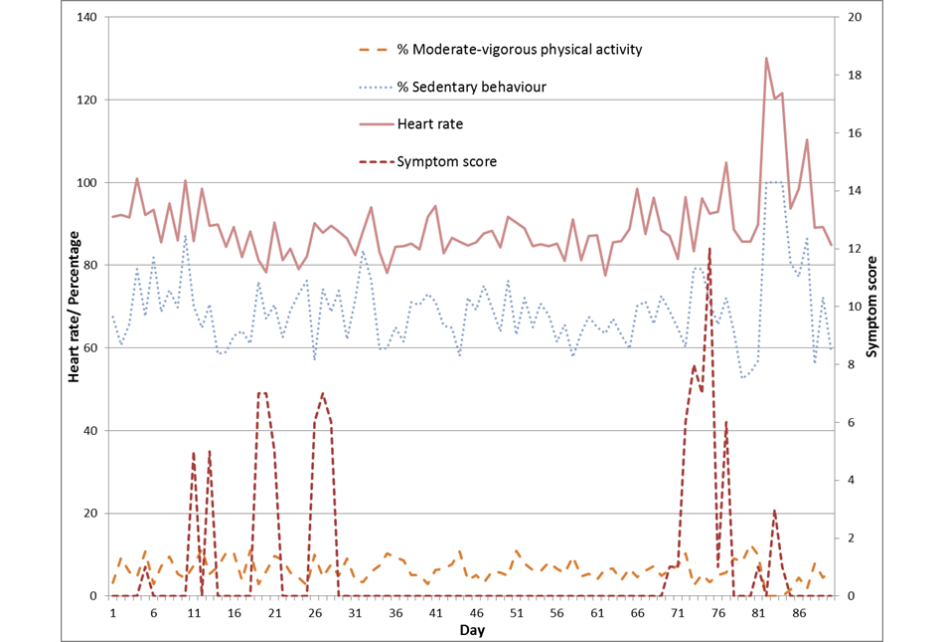
A participant’s heart rate, activity, and symptom scores over 90 days.

Patients had an average of 65.1 days of heart rate data available. For those days when data were available, there were on average 3170 heart rate recordings available per patient per day. Accelerometer data were available for 60.2 days on average. Due to the aforementioned technical issues, data were available for only 13 patients. For these patients, there were 592,000 accelerometer data points available per patient per day on average. After processing these to obtain sedentary behavior and moderate-to-vigorous physical activity, there were variations in patient activity (Table 2). An example of 1 patient’s daily average heart rate, percentage of sedentary behavior and physical activity, and questionnaire responses is shown in Figure 2.

**Table 2:** Summary of survey, heart rate, and activity data by patient.

Patient number	Survey		Heart rate		Activity		
	Score, mean (SD)	Days completed	Mean (SD)	Days of available data	MVPA^a^, mean %	Sedentary behavior, mean %	Days of available data
1	1.4 (2.7)	90	79.7 (8.7)	51	—	—	0
2	5.2 (4.8)	19	74.8 (6.1)	15	—	—	0
3	6.5 (4.8)	90	84.8 (5.4)	12	—	—	0
4	1.1 (2.5)	90	90.2 (9.5)	90	6.0	70.3	90
5	7.1 (5.4)	89	72.6 (7.8)	90	8.6	67.3	90
6	2.1 (3.8)	75	74.2 (5.8)	90	5.4	79.3	90
7	4.3 (4.0)	79	79.4 (10.7)	90	2.4	76.1	89
8	1.2 (2.1)	53	83.7 (16.5)	71	11.1	60.9	69
9	9.5 (4.7)	60	79.8 (10.2)	78	12.3	61.6	75
10	3.0 (4.3)	59	78.2 (11.3)	62	4.6	62.5	61
11	8.4 (4.8)	60	83.4 (7.4)	63	8.4	65.1	68
12	3.1 (3.7)	90	80.4 (6.4)	90	13.3	64.2	90
13	4.5 (4.0)	59	85.1 (8.2)	51	10.1	37.7	61
14	11.5 (4.8)	90	85.0 (5.7)	90	13.0	56.9	90
15	9.1 (3.4)	35	82.9 (15.8)	29	10.6	69.0	26
16	7.1 (5.3)	62	78.6 (4.0)	64	18.9	52.7	64
All	5.2 (5.3)	64.5	80.6 (10.4)	65.1	9.4	64.1	60.2

^a^MVPA: moderate to vigorous physical activity.

Fifteen participants provided feedback on issues concerning using a COPD wearable and what would make them regularly use a COPD wearable. Those who dropped out expressed privacy issues with the study specifically recording audio and others noted technology issues with the smartwatch system that made them want to stop participating. There were concerns with the bulky smartwatch, and some expressed desire for a thinner, lighter, and more stylish wristband.

Participants expressed that they wanted an app and a device that could provide more feedback. This feedback would include information about themselves in terms of their heart rate, coughing data, and oxygen saturation. Participants were also interested in gaining knowledge by accessing COPD educational material, breathing exercises, and physical activity exercises. Some participants also liked the idea of their health care provider being alerted if their symptoms were getting worse while others worried their health care provider would find it a nuisance.

## Discussion

### Principal Findings

We conducted a feasibility study of smartwatches in people with COPD and found that they will wear and maintain smartwatches that passively monitor audio, heart rate, and physical activity data almost continuously. Thus, smartwatches appear to be a viable platform for the intensive sensor data collection that may be required to detect AECOPDs early.

The frequency of sensor data collected was much greater than the frequency of other previously described interventions, as we collected data for 2 minutes out of every 10 minutes. While most previous COPD telemonitoring studies collected data daily [10], the one study with more frequent collection used a custom wristband that was designed to perform 5 measurements of their oxygen saturation, heart rate, body temperature, and physical activity every 3 hours [17]. However, they obtained on average only 4 recordings per patient day, and data were available for only 60% of patient days, seemingly due to lack of use of their system.

We found that there were a high percentage of patients who were not interested in participating. While telemonitoring studies have not consistently reported their recruitment rate [18,19], our recruitment rate was lower than the rate found in the custom COPD wristband study (16% vs 57%) and our dropout rate was higher (43% vs 22%) [17]. This may have been due to privacy concerns of recording audio or the differences in recruitment settings, as one of our sources of patients was hospitalized patients who were often too sick to participate. We heard from participants that to increase enrollment, we should provide patients with feedback about their activity, their heart rate, and how to better manage their COPD because they did not just want a passive monitoring device.

We found that we can collect symptom data through the daily symptom card, but this was not consistently completed even in our motivated participants. The availability of symptoms surveys that our patients provided was similar to the literature (70% in our study vs 77% in a similar study) [20]. This may support the approach for data collection through passive sensors because it may be problematic to reliably obtain symptom surveys from patients with COPD on a long-term basis [13].

We found gender differences in recruitment with more men agreeing to participate and more men completing the study. This may be due to the large size of the smartwatches as several people commented on the bulkiness of the smartwatches. These gender differences should be explored further to determine if there are other factors which may deter women from using a wearable to help manage their COPD.

### Limitations

There were significant limitations to our study. Many patients approached were not interested in participating. While this may partially be due to being part of a research study, it does call into question the generalizability of our results. We also had significant technical issues that negatively affected the retention of users and availability of data. Specifically, we found that the Android Wear platform was not a stable platform due to constant updates and operating system changes. Unfortunately, we were unable to definitively determine how much physiologic data were unavailable due to technical issues as opposed to not charging the device. As well, while the daily symptom score has been previously correlated with AECOPDs, we found a large variation in how patients would report symptoms. Some reported symptoms that were above their baseline, but some appeared to report any symptoms despite instruction. While the daily diary cards have been validated on paper [14], their use on a mobile phone app should be validated prior to further use. Finally, we collected heart rate and activity data but their accuracy is unclear because the smartwatches did not contain medical grade sensors.

Now that we have confirmed that some patients with COPD will use wearable devices that can obtain physiologic data, our next step is to engage patients in further depth to determine what they would like to see in a wearable device to help manage their COPD. This should increase the uptake and retention of both men and women with COPD. Further work would also include the validation of physiologic signals including cough detection, heart rate, and physical activity in predicting early AECOPDs.

### Conclusion

We found that patients with COPD will wear and maintain smartwatches that passively monitor audio, heart rate, and physical activity, and we were able to reliably capture near-continuous patient data. Further work is necessary to increase acceptability and improve the patient experience.

## Abbreviations

AECOPD: acute exacerbation of chronic obstructive pulmonary disease
AGE-WELL NCE: Aging Gracefully across Environments using Technology to Support Wellness, Engagement and Long Life—Networks of Centres of Excellence
COPD: chronic obstructive pulmonary disease
MVPA: moderate to vigorous physical activity
